# The Programming Power of the Placenta

**DOI:** 10.3389/fphys.2016.00033

**Published:** 2016-03-14

**Authors:** Amanda N. Sferruzzi-Perri, Emily J. Camm

**Affiliations:** Department of Physiology, Development and Neuroscience, University of CambridgeCambridge, UK

**Keywords:** placenta, fetus, programming, maternal environment, pregnancy, nutrient transport

## Abstract

Size at birth is a critical determinant of life expectancy, and is dependent primarily on the placental supply of nutrients. However, the placenta is not just a passive organ for the materno-fetal transfer of nutrients and oxygen. Studies show that the placenta can adapt morphologically and functionally to optimize substrate supply, and thus fetal growth, under adverse intrauterine conditions. These adaptations help meet the fetal drive for growth, and their effectiveness will determine the amount and relative proportions of specific metabolic substrates supplied to the fetus at different stages of development. This flow of nutrients will ultimately program physiological systems at the gene, cell, tissue, organ, and system levels, and inadequacies can cause permanent structural and functional changes that lead to overt disease, particularly with increasing age. This review examines the environmental regulation of the placental phenotype with particular emphasis on the impact of maternal nutritional challenges and oxygen scarcity in mice, rats and guinea pigs. It also focuses on the effects of such conditions on fetal growth and the developmental programming of disease postnatally. A challenge for future research is to link placental structure and function with clinical phenotypes in the offspring.

In the late 1980's, the epidemiologist and clinician David Barker found an unexpected link between low birth weight, an indicator of poor nutrition during pregnancy, and an increased risk of heart disease in adulthood. Barker and colleagues hypothesized over a series of studies that an adverse fetal environment followed by plentiful food in the postnatal period may lead to adult chronic disease (Barker and Osmond, 1986; Barker et al., 1989, 1993). Opponents of Barker's hypothesis argued that early nutrition was inferred indirectly from fetal and infant growth, and that most of the epidemiological studies were also vulnerable to confounding factors, particularly social class, that influence both the intrauterine and adult environments. Epidemiological studies arising from the Dutch famine of 1944 were a key test of Barker's hypothesis. Babies exposed to the famine during gestation were found to more likely to suffer from obesity, type 2 diabetes, cognitive deficits and heart disease, as well as die earlier as adults (Barker, 2004a,b,c; de Rooij et al., 2010). Whilst Barker's concept was initially controversial, an explosion of studies across a variety of human populations and experimental animals exposed to various insults during gestation showed that, in addition to metabolic and cardiovascular diseases, other chronic conditions such as cancer, allergies, asthma and neurocognitive disorders are also predetermined by the environment experienced in the womb and observed across the normal continuum of birth weights (McMillen and Robinson, 2005; Hanson and Gluckman, 2008; Rees et al., 2008; Susser and St Clair, 2013). Thus, there has been a revolutionary shift in thinking about how human qualities, such as appetite and metabolism, intelligence, temperament and susceptibility to disease are determined, and when they begin to develop. Moreover, there has been a drive to better identify the factors controlling intrauterine growth that are important in early-life programming of health in later life.

The main determinant of intrauterine growth is the placental supply of maternal nutrients and oxygen to the fetus. Indeed, in many species fetal weight near term and/or birth weight positively correlate with placental weight, and these associations have been suggested to serve as an indicator of the placental capacity to support fetal growth (Fowden et al., 2009). However, during pregnancy the placenta is exposed to a variety of environmental signals which can alter placental formation, and thus, the association of fetal weight to placental weight. Total food intake and macro- and micro-nutrient composition can vary during pregnancy due to seasonal changes in food availability, food fads or aversions and/or poor nutritional awareness about the harmful effects of cheap processed foods that are rich in sugar and fat (Vaughan et al., 2012a). Moreover, oxygen levels can be low in pregnancies at sea level due to cigarette smoking, maternal anemia, cord occlusion or poor placental vascularization, as well as in all pregnancies at high altitude (Zamudio, 2003; Hutter et al., 2010; Tissot van Patot et al., 2010). Attempts to emulate such conditions experimentally in animals, including mice, rats and guinea pigs, have shown that placental and fetal weights are altered (Table 1). In general, these studies show that the specific effects on placental and fetal weights appear to depend on the type of challenge (altered nutritional or oxygen availability, or both), the severity of the challenge and the duration and timing of the challenge in relation to formation of the placenta.

**Table 1:** **The effect of an environmental challenge on placental phenotype and fetal growth**.

**Manipulation**	**Species**	**Treated from**	**Fetal weight**	**Placenta**			**References**
				**Weight**	**Structural phenotype**	**Functional phenotype**	
**TOTAL CALORIE RESTRICTION**							
20%UN	Mouse	D3-D19	D16 ↔ D19 ↓13%	D16 ↓6% D19 ↓9%	D16 ↔Lz but ↓Jz and GlyT D19 ↓Lz (↓MBS vol, FC vol and SA) but ↔BT	D16 ↓Slc2a1 D19 ↑system A transport, Slc2a1, Slc38a2, Slc38a4	Coan et al., 2010
50%UN	Mouse	D2-12	D12 ND D19 ↔	D12 ↓15% D19 ↔	D12 ↓Jz ↑Lz/Jz ↓fetal blood space area ↓GlyT D19 ↔	D12 ↓Prl8a8	Schlitt and Schulz, 2012; Schulz et al., 2012; Harper et al., 2015
50%UN^*^	Mouse	D10-D19	D19 ↓48%	D19 ↓37%		D19 ↓glucose and system L amino transport ↑system A transport ↓GLUT3, LAT2, ↑GLUT1, Slc38a1, Slc38a2, FABP4, FABP5, apolipoproteins ↓pregnancy-specific glycoproteins	Ganguly et al., 2012; Chen et al., 2013
30%UN	Rat	D1-21	D21 ↓29%	D21 ↓25%		D21 altered expression of appetite regulating peptides in placenta	Caminos et al., 2008; Mayeur et al., 2012
35%UN	Rat	D15-20	D20 ↓18%	D20 ↔	D20 ↓antioxidant enzymes		Richter et al., 2009
50%UN	Rat	D5-20/21	D20/21 ↓15%	D20/21 ↓13%		D20/21 ↓placental system A uptake ↓utero-placental blood flow	Ahokas et al., 1981, 1983
50%UN	Rat	D10-20	D20 ↓25%	D20 ↓25%	D20 ↓Lz and Jz weights ↑apoptosis in both Lz and Jz	D20 ↓GLUT3, SNAT1, SNAT2 ↑SNAT4, GLUT1 ↓11bhsd2, ↑11bhsd1	Belkacemi et al., 2011a,b
50%UN	Rat	D14-20/21	D20/21 ↓11%	D20/21 ↓11%		D20/21 ↓utero-placental blood flow ↓GLUT3, ↔GLUT1 ↓11bhsd2	Ahokas et al., 1981, 1983; Lesage et al., 2001, 2002a
15%UN	Guinea pig	-D151-D61	D61 ↓26%	D61 ↓20%	D61 ↓FC and MBS	D61 ↓P-gp protein	Soo et al., 2012
10-30%UN	Guinea pig	-D28-D60	D35 ↓29% D60 ↓35%	D35 ↓20% D60 ↓30%	D35 ↓Jz ↔Lz, but ↓MBS, SA, ↑BT D60 ↔Jz ↓Lz, MBS, FC, SA, ↑BT		Sohlstrom et al., 1998a,b; Roberts et al., 2001
**LOW-PROTEIN DIET**							
16% vs. 20% protein	Mouse	D3-19	D16 ↔ D19 ↔	D16 ↑5% D19 ↑5%	D16 ↓Lz/Jz ratio D19 ↓Lz/Jz ratio	D16 ↑glucose transport, ↔system A transport, ↑Slc2a1 D19 ↔glucose transport, ↓system A transport, Slc38a4	Coan et al., 2011
8% vs. 20% protein	Mouse	D1-19	D15 ↓16% D19 ↓13%	D15 ↑10% D19 ↓27%	D15 ↔ D19 ↓MBS and FC length		Rutland et al., 2007
8% vs. 20% protein	Mouse	D3-19	D16 ↔ D19 ↓9%	D16 ↔ D19 ↑4%	D16 ↔ D19 ↔	D16 ↑glucose transport, ↔system A transport, ↑Slc38a2 D19 ↔glucose and system A transport, ↓Slc38a1, Slc38a4	Coan et al., 2011
8% vs. 20% protein	Rat	D1-21	D18/21 ↓12-20%	D18/21 ↑113%	D18 ↓Lz Vd, ↑Jz, ↑SA exchange, diffusing capacity	D18 ↓uterine blood flow	Itoh et al., 2002; Doherty et al., 2003; Liu et al., 2014
5% vs. 19% protein	Rat	D1-19	D19 ↔	D19 ↔	D19 ↓glyT, giT, Lz thickness		Rebelato et al., 2013
6% vs. 20% protein	Rat	D1-21	D14 ↓21.5 D18 ↓27 D21 ↓14%	D14 ↓25% D18 ↓12% D21 ↔	D14 ↓Lz and Jz vol D18 ↓Lz vol, ↔Jz vol Altered trophoblast stem cell and lineage markers D21 ↔Lz, ↓Jz	D14 ND D18 ↓Hsd17b2 D21 ↓Hsd17b2	Gao et al., 2012a,b, 2013
5% vs. 21% protein	Rat	D1-21	D21 ↓28%	D21 ↓24%		D21 ↓system A transport	Varma and Ramakrishnan, 1991
4% vs. 18% protein	Rat	D2-21	D15-19 ↔ D21 ↓21%	D15-19 ↔ D21 ↓12.5%		D19 and D21 ↓system A, system L transport capacity, ↓LAT1, LAT2, SNAT2 D21 ↓SNAT1 All ages ↔glucose transport, SNAT4	Jansson et al., 2006; Rosario et al., 2011
5% vs. 19% protein	Rat	D6-21	D20/21 ↓25%	D20/21 ↓28%		D20/21 ↓system A, system X${}_{AG}^{-}$ and system y^+^amino acid transport ↓glucose transport ↔ASC system amino acid transport ↓Slc1a1, Slc7a1	Malandro et al., 1996a; Rosso, 1977a,b
**HIGH-CALORIE DIETS**							
3.5x fat	Mouse	D1-D19	D19 ↔	D19 ↔		D19 ↑LPL protein and activity ↑CD36/FAT, VLDLr, FABP3, FABPpm	Qiao et al., 2015
2.9x fat	Mouse	-D98-D19	D19 ↑9%	D19 ↑6%	D19 ↑vascularization		Li et al., 2013
3.4x fat	Mouse	-D84-D18	D13-D16 ↔ D18 ↑12%	D13-D18 ↔		D13-D18 ↓Abcb1a, P-gp ↑digoxin transfer, IL-1β and TNF-α	Wang et al., 2015
5.3x fat^*^	Mouse	-D84-D19	D15 ↔ D19 ↓8%	D15-D19 ↔		D15 ↑Slc38a2 or ↑Slc38a4 D19 ↔	King et al., 2013
5.5x fat	Mouse	-D56-D16	D19 ↓23%	D19 ↓9%		D16 ↓11bhsd2 D19 ↓11bhsd1	Bellisario et al., 2015a,b
2.5x fat	Mouse	-D56-D18	D16 ↔ D18 ↑18%	D16 ↑12% D18 ↔	D16 and D18 ↓Lz and proliferation ↔Jz	D16 and D18 altered cytokine expression	Kim et al., 2014
3x fat	Mouse	-D56-D19	D19 ↑43%	D19 ↔		D19 ↑glucose and system A amino acid transport, Slc2a1, Slc38a2	Jones et al., 2008
5.4x fat	Mouse	-D49-D21	D21 ↑16%	D21 ↑10%		D21 ↑leptin, LPL	Mazzucco et al., 2013
6x fat	Mouse	-D42-D18	D18 ↑30%	D18 ↔		D18 ↑LPL, VLDLr, FABP3, FABPpm	Qiao et al., 2012
2.7x fat	Mouse	-D28-D19	D19 ↑15%	D19 ND		D19 ↔FATP1, 4, GLUT1, LPL	Rebholz et al., 2011
4.5x fat^*^	Rat	-D21-D18	D18 ↓11	D18 ↓23%	D18 ↔Lz ↓Jz	D18 ↑Lpl, Slc2a1, Slc2a4, Slc38a2, Cd36/FAT ↔Slc38a4	Reynolds et al., 2015
12x fat	Mouse	-D28-D19	D19 ↓8%	D19 ↓22%	D19 ↓trophoblast ↑endothelial apoptosis, oxidative stress		Liang et al., 2009a, 2010
6x fat^*^	Mouse	D1-15	D15 ↔	D15 ↑7%	D15 ↔Lz or vascularity	D15 ↓Slc22a1 ↑Slc22a2	Gallou-Kabani et al., 2010; Gabory et al., 2012
2.8x fat	Rat	-D112-D15	D15 ↓12% ↑fetal loss	D15 ↔	D15 ↑Lz vascularization but ↓smooth muscle actin ↑oxidative stress	D15 ↓remodeling of maternal spiral arteries	Hayes et al., 2012, 2014
5-6x fat	Rat	-D49-D21	D21 ↑7%	D21 ↔	D21 ↑mTOR signaling	D21 ↔system A, system L transport and LPL activity ↓SNAT1, ↔SNAT2, 4, GLUT1, 3, 9, FATP4, 6	Gaccioli et al., 2013
2.5x fat	Rat	D1-D21	D21 ↓5%	D21 ↔	D21 ↓Jz		Mark et al., 2011
3x fat 5x sugar diet	Mouse	D1-D19	D16 ↓9% D19 ↔	D16 ↓11% D19 ↓8%	D16 ↓Lz FC, ↑BT D19 ↓Lz, MBS, BT, SA and GlyT	D16 ↑glucose and system A transport, Slc2a3, Slc38a2 D19 ↔glucose and system A amino acid transport, ↑FATP1	Sferruzzi-Perri et al., 2013
3.5x fat diet plus fiber	Rat	D1-D20	D20 ↔	D20 ↑17%		D20 ↑Slc38a2, Slc2a1 and Slc2a3 ↔Slc38a4 or Slc7a1	Lin et al., 2011
Excess of 20% fructose in drinking water^*^	Rat	D1-D10	D21 ↔	D21 ↓7%	D21 ↔Lz or Jz or ratio		Vickers et al., 2011
Excess of 10% fructose in drinking water^*^	Rat	D1-D21	D21 ↔	D21 ↓7%	D21 ↔Lz or Jz or ratio	D24 ↓Slc2a1, ↑Slc38a2 ↔Slc2a3 Uterine vascular responsiveness impaired	Alzamendi et al., 2012; Lineker et al., 2015
**HYPOXIA**							
13% hypoxia	Mouse	D11-16	D16 ↔	D16 ↔	D16 ↑Lz, MBS, trophoblast vol, SA exchange	D16 ↔glucose or system A transport, Slc2a, Slc38a	Higgins et al., 2015
13% hypoxia	Mouse	D14-19	D19 ↓5%	D19 ↔	D19 ↑FC Vd ↓BT	D19 ↑placental glucose transport ↔system A transport ↑Slc38a1	Higgins et al., 2015
12% hypoxia^*^	Mouse	D14-19	D19 ↓6.5%	D19 ↔	D19 ↓Lz blood space ↑tissue	D19 ↓Slc2a1, 11bsd2 ↑Slc38a1 ↔Slc2a3 ↓angiotensinogen	Cuffe et al., 2014a,b
10.5% hypoxia	Mouse	D11-19	D19 ↓36%	D19 ↔		D18 altered uterine artery function	Rueda-Clausen et al., 2014
10% hypoxia	Mouse	D14-19	D19 ↓21%	D19 ↔	D19 ↓Lz Vd, ↑Jz Vd ↓MBS vol and SA ↑trophoblast vol, BT	D19 ↓system A transport ↔glucose transport Altered uterine artery vasoreactivity	Higgins et al., 2015; Skeffington et al., 2015
13% hypoxia^*^	Mouse	D1-D19	D19 ↓12%	D19 ↑10%	D19 ↑maternal arterial and venous blood space		Matheson et al., 2015
13% hypoxia	Rat	D6-D20	D20 ↔	D20 ↑5%	D20 ↔Lz, Jz vol or Vd ↑oxidative stress		Richter et al., 2012
11% hypoxia	Rat	D7-14	D14 ND	D14 ↑25% total area	D14 ↑Jz and Lz (greater expansion of Jz vs. Lz)	D14 ↑maternal uterine vascular remodeling ↑prolactin-like genes	Ho-Chen et al., 2006; Rosario et al., 2008
12% hypoxia followed by 14% hypoxia	Guinea pig	D15-64	D64 ↓24%	D64 ↓31%	D64 ↓diffusion capacity, SA exchange, ↑BT		Bacon et al., 1984
12% hypoxia	Guinea pig	D15-64	D64 ↔	D64 ↔	D64 ↑diffusion capacity, vascular vol, ↔SA, ↓BT		Bacon et al., 1984
12% hypoxia	Guinea pig	D18-63	D63 ↓30%	D64 ↔	D64 ↑diffusing capacity		Gilbert et al., 1979
**IRON RESTRICTION**							
Iron restriction	Rat	-D21-D21	D21 ↓11%	D21 ↓18%			Crowe et al., 1995
Iron restriction	Rat	-D14-D21	D21 ↓15%	D21 ↑8%	D21 ↑Lz vol, total SA ↓fetal capillary length and SA		Lewis et al., 2001a
Iron restriction	Rat	-D7-D21	D21 ↓20%	D21 ↔	D21 ↔Lz vol, total SA ↓fetal capillary length and SA		Lewis et al., 2001a
**UTERINE SUBSTRATE SUPPLY**							
Uterine ligation	Rat	D14	D19 ↓20%	D19 ↓19%	D19 ↑expression of apoptotic genes	D19 ↑expression of prolactin-like genes	Alexander et al., 2001; George et al., 2014
Uterine ligation	Rat	D18 or 19	D20/22 ↓15-25%	D20/22 ↔ or ↓10-15%	D20/22 ↓placental blood flow ↑placental diameter ↔Lz area or vol	D20/22 ↓GLUT1 ↔GLUT3	Gilbert and Leturque, 1982; Das et al., 1998; Reid et al., 1999; Wlodek et al., 2005

*AA, amino acid; Abcb1a, multidrug resistance protein; 11bhsd, 11 beta-hydroxysteroid dehydrogenase; BT, barrier thickness; CD36/FAT, fatty acid translocase; D, day; FABP, fatty acid binding protein; FATP, fatty acid transport protein; FC, fetal capillaries; GiT, trophoblast giant cells; GLUT/Slc2a, glucose transporter; GlyT, trophoblast glycogen cells; Jz, junctional zone; LAT, L-type amino acid transporter; LPL, lipoprotein lipase; Lz, labyrinthine zone; MBS, maternal blood space; ND, not determined; P-gp, phosphoglycoprotein; SA, surface area; SNAT/Slc38a, Sodium-coupled neutral amino acid transporter; Vd, volume density; VLDLr, very low density lipoprotein receptor; vol, volume; y+ system*.

* *Effects depend on conceptus sex*.

*Gestational age: mouse ~20 days, rats ~23 days, guinea pigs ~70 days*.

## Regulation of fetal and placental weights by the maternal environment

Reducing maternal food intake by 15–50% for the majority of pregnancy in mice and rats, or prior to and during pregnancy in guinea pigs, results in fetal and placental weights that are, on average, 22–26% lower than *ad libitum* controls (Ahokas et al., 1981, 1983; Sohlstrom et al., 1998a,b; Roberts et al., 2001; Caminos et al., 2008; Coan et al., 2010; Ganguly et al., 2012; Mayeur et al., 2012; Schlitt and Schulz, 2012; Schulz et al., 2012; Soo et al., 2012; Table 1). In the guinea pig, the capacity of the mother to deliver nutrients to the fetus is further impaired if maternal nutrient reserves are depleted by undernutrition prior to conception (Sohlstrom et al., 1998a,b, 2001; Roberts et al., 2001). Thus, poor intrauterine growth is likely to be of early onset in this model. When assessing the studies summarized in Table 1, the greatest reductions in feto-placental growth are reported for pregnant mice, and are also observed if the nutrient restriction occurred from mid-gestation (Ganguly et al., 2012). Fetal growth rate for the mouse is much higher than for most species, including rats and guinea pigs (Fowden and Moore, 2012), and may therefore be more sensitive to changes in nutrient supply. Low-protein isocalorific diets for most of pregnancy also reduce fetal weight by ~18% near term in mice and rats, highlighting the importance of dietary protein for fetal tissue accretion (Rosso, 1977a,b; Varma and Ramakrishnan, 1991; Malandro et al., 1996a; Doherty et al., 2003; Jansson et al., 2006; Rutland et al., 2007; Vieira-Filho et al., 2009; Coan et al., 2011; Rosario et al., 2011; Gao et al., 2012a,b, 2013; Liu et al., 2014; Table 1). However, depending on the degree of protein deprivation and the source of the extra carbohydrate used to maintain the calorific content of the diet, low-protein diets have been associated with reduced, increased or unchanged placental weight in near-term rodents (Table 1). If global undernutrition or protein deprivation occurs solely in the second half of pregnancy, fetal weight is reduced even though placental weight may be unchanged (Franko et al., 2009; Gheorghe et al., 2009; Richter et al., 2009; Higgins et al., 2015; Table 1). This suggests that gross growth of the rodent placenta exhibits a degree of resilience to short-term nutritional insults once it has formed. There may also be catch-up growth of the placenta in late gestation if the nutritionally-deprived dams are returned to *ad libitum* feeding of the control diet. For instance, the effect of global undernutrition to reduce placental weight was mitigated by *ad libitum* feeding of the dam in late pregnant mice (Harper et al., 2015). This suggests alterations in the placenta caused by nutrient restriction in early pregnancy could be reversible.

Excess calories delivered through diets high in fat, simple sugars, or both, for months prior to and during pregnancy have varying effects on feto-placental growth in mice and rats. Diets with 2.5 to 6-times the fat content of control chow tend to increase fetal weight, often in the absence of changes in placental weight (Jones et al., 2008; Rebholz et al., 2011; Qiao et al., 2012; Gaccioli et al., 2013; Li et al., 2013; Mazzucco et al., 2013; Dahlhoff et al., 2014; Kim et al., 2014; Wang et al., 2015). However, when dietary fat content exceeds 6-times control values, or high-fat diets are consumed for several months prior to conception, fetal and placental weights are often decreased (Taylor et al., 2003; Liang et al., 2009a, 2010; Jungheim et al., 2010; Hayes et al., 2012, 2014; King et al., 2013; Bellisario et al., 2015a,b; Reynolds et al., 2015; Wu et al., 2015). The reduction in conceptus growth in these studies may be secondary to the systemic inflammatory state in, and/or a greater competition for resources by, the pre-conceptionally chronically obese dam. When high-fat diets are fed from day 1 of pregnancy, fetal weight is unaltered or marginally reduced, with both increased and reduced placental weights reported depending on whether simple sugars or fiber were additionally consumed in excess (Gallou-Kabani et al., 2010; Lin et al., 2011; Mark et al., 2011; Gabory et al., 2012; Sferruzzi-Perri et al., 2013; Qiao et al., 2015). In rodents, increasing the proportion of energy intake from sugar, either by adding fructose to their drinking water or by supplying sweetened condensed milk, has been associated with both unchanged and reduced fetal and placental weights depending on the length of exposure (before, during or part of pregnancy) and whether gross maternal food intake was reduced (Holemans et al., 2004; Vickers et al., 2011; Gray et al., 2013; Mukai et al., 2013; Lager et al., 2014). Rodents exhibit tight homeostatic control of their energy intake (Keesey and Hirvonen, 1997), and the contribution of the reduced protein and/or micronutrient intake to the effects of calorie-dense diets on feto-placental phenotype requires clarification (Armitage et al., 2004). Embryo transfers between mice fed a control or high-fat diet prior to pregnancy have shown that the effect of pre-conception obesity and an adverse metabolic milieu on feto-placental growth is not mitigated by a control diet during gestation, indicating permanent effects on development (Sasson et al., 2015). Maternal oocyte quality and conceptus metabolism are altered in rodents that are obese or fed diets with altered nutrient composition pre-gestationally (Minge et al., 2008; Mitchell et al., 2009; Igosheva et al., 2010; Jungheim et al., 2010; Luzzo et al., 2012). Moreover, maternal protein deprivation for as short as 4 days post-conception is sufficient to influence the allocation of trophectoderm and inner cell mass cells within the rodent blastocyst, as well as trophoblast cell proliferation and differentiation with consequences for subsequent development of the definitive placenta and the fetus (Kwong et al., 2000, 2006; Watkins et al., 2008, 2015). Conceptus weight in the latter part of gestation therefore, reflects the availability of specific nutrients before and during pregnancy; from the earliest stages of development and cell lineage determination through to the metabolic and morphological modifications of the fetus and placenta that occur toward term.

Reduced oxygen availability induced by housing animals in chambers where the inflow of oxygen is displaced by nitrogen, also affects conceptus growth. In mice, rats and guinea pigs, fetal growth is reduced in a severity-dependent fashion, suggesting fetal growth is highly sensitive to alterations in atmospheric oxygen content (Table 1). For instance, fetal weight is unaltered by 16% inspired maternal oxygen, reduced on average by ~10% with 12–13% oxygen and restricted by >22% in association with fetal loss if atmospheric oxygen drops to < 11% in pregnant mice, rats and/or guinea pigs (Gilbert et al., 1979; Bacon et al., 1984; Lueder et al., 1995; Richter et al., 2012; Cuffe et al., 2014a; Rueda-Clausen et al., 2014; Higgins et al., 2015; Matheson et al., 2015). In mice and guinea pigs, fetal growth is most adversely affected if the hypoxic challenge occurs in the last third of pregnancy when fetal growth is maximal. In contrast to the fetus, maternal inhalation hypoxia does not reduce placental weight and can cause placentomegaly if the hypoxic challenge commences within the first third of gestation. Placentomegaly may be secondary to a hypoxia-induced increase in trophoblast invasion of the maternal decidua (Alam et al., 2007; Rosario et al., 2008; Chakraborty et al., 2011) as this process mediates both circumferential expansion of, and maternal blood flow to, the placenta. Pregnant rodents and guinea pigs exposed to severe hypoxic atmospheres have been reported to reduce their food intake, and therefore the resultant effects on the feto-placental phenotype may be partly attributable to maternal hypophagia (Bacon et al., 1984; Camm et al., 2010, 2011; Higgins et al., 2015; Matheson et al., 2015). In rats, anemia induced by nutritional deficiency of iron prior to pregnancy reduces fetal weight, with variable effects on placental weight depending on the length of exposure and strain of rat (Crowe et al., 1995; Lewis et al., 2001a). In rats and guinea pigs, when both nutrient and oxygen availability to the conceptus are reduced by uterine artery ligation, fetal and placental growth tend to be restricted to a greater degree than observed with hypoxia and maternal food deprivation alone (Gilbert and Leturque, 1982; Das et al., 1998; Reid et al., 1999; Alexander et al., 2001; Carter et al., 2005; Wlodek et al., 2005; Turner and Trudinger, 2009; George et al., 2014). To date, only one study has been performed in the mouse, and showed reduced fetal growth in the absence of a change in placental weight near term when uterine blood flow is reduced by 40% (Intapad et al., 2014). Thus, environmentally-induced alterations in the ability of the mother to supply nutrients and oxygen both prior to and during pregnancy, affect fetal and placental growth.

Generally, an adverse maternal environment will have a greater influence on fetal than placental growth (Table 1), suggesting that the placenta may be spared over other organs (Vaughan et al., 2012a). However, in cases of environmentally-induced fetal overgrowth, fetal changes occur without a change in placental weight, suggesting enhanced placental efficiency in supporting growth (Jones et al., 2008; Rebholz et al., 2011; Gaccioli et al., 2013; Li et al., 2013; Mazzucco et al., 2013; Dahlhoff et al., 2014; Kim et al., 2014; Wang et al., 2015). When the growth kinetics of the conceptus in resource-limited rodents pregnancies have been assessed, it appears that alterations in placental weight occur before noticeable retardation of fetal growth (Coan et al., 2010; Sferruzzi-Perri et al., 2011; Kim et al., 2014). For instance, fetal growth is maintained until at least day 16 in undernourished pregnant mice, even though placental weight is reduced at this gestational age (Coan et al., 2010; Sferruzzi-Perri et al., 2011). Furthermore, with some nutritional manipulations the placenta is more adversely affected than the fetus near term (Rutland et al., 2007; Liang et al., 2009a, 2010; Sferruzzi-Perri et al., 2013; Reynolds et al., 2015). Indeed, in mice fed obesogenic diets high in sugar and fat during pregnancy, fetuses attain a normal body weight near term despite the persistence of reduced placental weight and increased maternal adiposity (Sferruzzi-Perri et al., 2013). Thus, environmental challenges can affect the relationship of fetal weight to placental weight, and suggest that in some instances, the placenta may adapt its capacity to optimize fetal growth and survival in the prevailing conditions *in utero* (Fowden et al., 2009). Such changes in placental phenotype could be exerted at a morphological and/or functional level.

## Regulation of placental structure by the maternal environment

Changes in placental capacity to support fetal growth could be generated by environmentally-induced alterations in the gross structure of the placenta, conferring a functional advantage. In rodents and guinea pigs, the placenta is organized into discrete regions that function predominately in materno-feto transport and hormone production; the labyrinthine zone and junctional/interlobium zone, respectively. These zones have a different tempo of development and maturation. In some environmental manipulations, both regions are proportionately altered and track with near-term placental weight (Wlodek et al., 2005; Belkacemi et al., 2011a; Table 1). However, in many studies the two regions respond differently to the environmental manipulation (Table 1). For instance, in rats the formation of the junctional zone is specifically reduced by a maternal high-fat diet and in undernourished mice and guinea pigs the volume of junctional/interlobium zone is decreased earlier in gestation than that of the labyrinthine zone (Roberts et al., 2001; Coan et al., 2010; Mark et al., 2011; Sferruzzi-Perri et al., 2011; Schulz et al., 2012). These studies suggest that there may be an active preservation of placental transport function, even at the expense of placental endocrine region formation, to optimize substrate delivery to the fetus at particular phase/s of development. However, in mice fed a low-protein diet or calorific-dense diets with altered protein content, this labyrinthine zone sparing is not observed and the labyrinthine zone is more adversely affected than the junctional zone, which would limit the provision of nutrients to the fetus (Doherty et al., 2003; Coan et al., 2011; Rebelato et al., 2013; Sferruzzi-Perri et al., 2013; Kim et al., 2014).

The severity of maternal oxygen deprivation also affects the gross morphology of the placenta. Maternal iron-depletion or 12–13% inhalation hypoxia in the last third of pregnancy selectively expanded the labyrinthine zone, which would optimize materno-fetal substrate delivery (Lewis et al., 2001a; Cuffe et al., 2014a; Higgins et al., 2015). Conversely, if maternal inspired oxygen is lowered to 10%, the labyrinthine zone is reduced with a concomitant increase in the volume density of the junctional zone (Rosario et al., 2008; Higgins et al., 2015). Switching conceptus metabolism from aerobic to anaerobic by inhibiting mitochondrial oxidation early in blastocyst development also alters placental formation in rats near term (Wakefield et al., 2011). Environmentally-induced changes in the gross architecture of the placenta have been linked to altered expression of genes and proteins involved in proliferation, apoptosis, oxidative stress and cell lineage differentiation (Kwong et al., 2000; Gheorghe et al., 2007, 2009; Liang et al., 2009a, 2010; Richter et al., 2009; Belkacemi et al., 2011b; Chen et al., 2013; Gao et al., 2013; Kim et al., 2014; Matheson et al., 2015; Watkins et al., 2015). Thus, regions in the placenta dedicated to transport and endocrine function appear differentially sensitive to changes in the maternal supply of nutrients and oxygen. Such changes depend on the type and timing of the insult, and the species examined.

Maternal environmental manipulations can also alter the ultrastructure of the placental transport region. Following maternal caloric restriction in the guinea pig, and severe maternal oxygen deprivation (10%) in the mouse, the thickness of the trophoblast barrier between the maternal and fetal circulations is increased, and the surface area for exchange and density of fetal capillaries and/or maternal blood spaces diminished (Roberts et al., 2001; Soo et al., 2012; Hvizdosova-Klescova et al., 2013; Higgins et al., 2015). Placental fetal capillary length and surface area is reduced, and the integrity and normal developmental architectural rearrangements of the fetal vasculature and maternal blood spaces decreased, following exposure to low-iron or low-protein diets, or global nutrient restriction (Lewis et al., 2001a; Rutland et al., 2007; Schulz et al., 2012). Such perturbations in the placental transport region will further limit substrate transfer, particularly by flow-limited passive diffusion processes (Fowden et al., 2006). There are, however, also beneficial changes that occur in placental morphology following a maternal environmental challenge (Table 1). For instance, the surface area for exchange and the diffusing capacity are increased in the overgrown placenta of protein-deprived rat dams (Doherty et al., 2003). Moreover, the interhemal barrier is thinner, and the exchange surface area and proportions of maternal and/or fetal blood compartments within the placenta are increased in mice and guinea pigs exposed to 12–13% hypoxia (Gilbert et al., 1979; Bacon et al., 1984; Higgins et al., 2015; Matheson et al., 2015). These structural modifications will increase the placental capacity for materno-fetal diffusion and suggest placental adaptation. There are also gestational-age dependent alterations in placental fine architecture with maternal environmental manipulation that track with changes in fetal growth. For instance, the placental interhemal membrane barrier is thicker and vascularization reduced on day 16 of gestation when fetuses are growth restricted, in mouse dams consuming a diet high in both sugar and fat (Sferruzzi-Perri et al., 2013). However, by day 19 of pregnancy, these structural changes improve (thinner barrier) or are restored to control values (vascularization), in line with the normalization of fetal weight (Sferruzzi-Perri et al., 2013). In many of the environmentally-manipulated pregnancies, there are also changes in uterine vascular responsiveness and/or trophoblast remodeling of the maternal spiral arteries which have implications for blood flow and the delivery of substrates to the placenta (Ahokas et al., 1981, 1983; Itoh et al., 2002; Taylor et al., 2003; Alam et al., 2007; Rosario et al., 2008; Chakraborty et al., 2011; Hayes et al., 2012, 2014; Rueda-Clausen et al., 2014; Lineker et al., 2015; Skeffington et al., 2015). Thus, maternal nutritional challenges and oxygen availability affect the composition of the materno-fetal interface, which will have ramifications for placental substrate transport and thus fetal growth.

## Regulation of placental transport function by the maternal environment

Along with oxygen, glucose, amino acids and fatty acids represent essential nutrients for fetal growth. The placenta transports these substrates to the fetus by passive diffusion, transporter-mediated processes and endocytosis-exocytosis (Sibley et al., 1997; Duttaroy, 2009). Molecules like oxygen, carbon dioxide and urea traverse the placenta by passive diffusion. Glucose and lactate are transported down their concentration gradient by facilitated diffusion using transporter proteins embedded in the plasma membrane, without a requirement for additional energy. Amino acids are actively transported against their concentration gradient, using both membrane transporter proteins and the input of additional energy. Materno-fetal transfer of fatty acids is less understood although it involves the release of fatty acids from maternal triglycerides using lipases and the coordinated action of both cytoplasmic and membrane carrier proteins for diffusion. In addition to morphological characteristics of the placenta, transporter-mediated processes are influenced by the expression, localization, affinity and activity of specific transporters in the placental plasma membranes as well as the materno-fetal concentration gradient across the placenta (Hay, 1995; Jansson and Powell, 2006). Changes in any of these placental parameters can, therefore, affect fetal fuel acquisition and growth with consequences for adult health and disease (Fowden et al., 2008).

The maternal environment modifies placental glucose transport capacity (Table 1). Trans-placental transfer of glucose *in vivo* is unaltered by under-nourishing pregnant mice to 80% of the *ad libitum* daily intake. However, when maternal total food intake is reduced to 50% of *ad libitum* values, trans-placental transfer of glucose near term is diminished by ~40% (Coan et al., 2010; Ganguly et al., 2012). In rats, glucose transfer is also diminished if the protein content of the maternal diet is as low as 5% (Rosso, 1977a), but unaltered by a 4% protein diet (Jansson et al., 2006). Reductions in glucose transfer will further deprive the fetus of this important metabolic fuel and likely exacerbate the effects of maternal dietary manipulations on fetal growth. However, in mice trans-placental glucose transfer is increased in late gestation when the protein deprivation is less severe, with 8 and 16% protein diets (Coan et al., 2011), suggesting that the placenta may be trying to compensate for a deficit in the supply of another nutrient. In rodents near term, placental expression of the glucose transporter Slc2a3/GLUT3 is typically reduced by maternal undernutrition, whilst expression of Slc2a1/GLUT1 appears to increase under nutrient scarcity (Lesage et al., 2002a; Coan et al., 2010, 2011; Belkacemi et al., 2011a; Ganguly et al., 2012). Combined, these data indicate the differential responsiveness of specific transporter subtypes in the placenta following maternal nutritional manipulation. Excess dietary calories can also affect glucose transport. For instance in mice, materno-fetal clearance of glucose is increased near term by diets high in fat alone, as well as on day 16 of gestation in those also consuming excess sugar (Jones et al., 2008; Sferruzzi-Perri et al., 2013). Such changes were associated with increased expression of Slc2a1/GLUT1 and Slc2a3/GLUT3 by the placenta, respectively. In rats, high-fat diets with an altered fiber content during pregnancy also enhance placental expression of Slc2a1/GLUT1 and/or Slc2a3/GLUT3 just prior to delivery (Lin et al., 2011), whereas placental Slc2a1/GLUT1 is instead reduced by high dietary sugar intake (Alzamendi et al., 2012). Whether *in vivo* placental glucose transfer is altered in these studies however, is unknown. Exposure to 13% inspired oxygen for 5 days from pregnancy day 14, also increases placental uptake and clearance of glucose in mice, which suggests that glucose becomes a more important metabolic substrate in feto-placental tissues when oxygen availability is limited near term (Higgins et al., 2015). Certainly, in hypoxic pregnant rats, glucose uptake and lactate production by the fetuses is increased, suggesting glycolytic metabolism in the fetuses which would maintain the supply of energy for fetal growth (Lueder et al., 1995). However, 10% inspired oxygen does not affect placental uptake and trans-placental glucose transport in mice on day 19 and results in severe fetal growth restriction (Higgins et al., 2015). In rats where both nutrient and oxygen delivery to the conceptus is restricted by maternal uterine artery ligation, Slc2a1/GLUT1 is selectively reduced in the placenta (Das et al., 1998). Thus, placental glucose transfer is sensitive to maternal environmental manipulation but the specific response elicited is insult, severity and species-dependent, and may optimize or further compromise conceptus growth in the prevailing condition.

The capacity of the placenta to deliver amino acids to the fetus is also modified by the maternal environment (Table 1). In rats consuming diets that contain 4–5% protein, the activity of the Systems A, L, X^−AG^ and y^+^ are diminished near term, in association with altered expression of Slc38a1/SNAT1, Slc7a5/LAT1, Slc7a8/LAT2 and Slc1a1/EAAC1, and Slc7a1/CAT1 amino acid transporters (Rosso, 1977b; Malandro et al., 1996a,b; Jansson et al., 2006; Rosario et al., 2011). These functional perturbations will further limit the supply of neutral, anionic and cationic amino acids to the fetus and contribute to the intrauterine growth restriction reported. Indeed, in this species down-regulation of System A and L amino acid transport precedes the onset of fetal growth restriction in dams fed the 4% protein diet (Jansson et al., 2006), and partial inhibition of System A activity *in vivo* induces fetal growth restriction in late gestation (Cramer et al., 2002), signifying the importance of amino acid transport for prenatal development. Placental System A transporter activity is also diminished near term in mice fed a 16% protein diet, but is instead unaltered by a more severe reduction to 8% dietary protein (Coan et al., 2011). The expression of Slc38a4/SNAT4 is reduced by both protein manipulations in the mouse placenta near term, although placental expression of the higher affinity System A transporter, Slc38a2/SNAT2 is increased in mice fed the 8%, but not 16% protein diet compared to controls (Strakovsky et al., 2010; Coan et al., 2011). Altering the calorie intake of rodents also affects amino acid transport capacity. Feeding mice dams 50% of *ad libitum* food intake from mid-gestation reduces placental System L activity just prior to term, in line with decreased Slc7a8/LAT2 abundance. However, in these mice (50% undernutrition) and those fed 80% of *ad libitum* food intake, transfer of amino acids via System A is adaptively increased near term and is coupled with enhanced placental Slc38a1/SNAT1 and/or Slc38a2/SNAT2 expression (Coan et al., 2010; Sferruzzi-Perri et al., 2011; Ganguly et al., 2012). Mice consuming high-calorie fat diets with and without excess sugar also show enhanced placental System A activity in association with elevated Slc38a2/SNAT2 expression and accelerated fetal growth (Jones et al., 2008; Sferruzzi-Perri et al., 2013). Slc38a2/SNAT2 expression is also increased in the placenta of rats fed high sugar or fat diets (Lin et al., 2011; Alzamendi et al., 2012). However, in another rat study, Slc38a1/SNAT1 abundance was decreased by a high-fat diet with no change in placental System A capacity *in vivo* (Gaccioli et al., 2013). Capacity of the placenta for amino acid transport is also responsive to changes in maternal oxygen availability. Inhalation hypoxia at 10%, but not 13%, adversely affects placental System A transport *in vivo* in near-term mice. This alteration appears to be related to the reduction in maternal food intake and the inability of the placenta to maintain an energy supply for the active transport of amino acids, specifically in the 10% hypoxia group (Higgins et al., 2015). Thus, the fetal provision of nutrients may also be decreased as a secondary effect of reduced maternal oxygen availability on placental transport capacity. Indeed, fetal concentrations of several amino acids are altered in anemic rats (Lewis et al., 2001b). Further work is required to determine the environmental regulation of other amino acid transporters in the placenta that have been implicated in facilitating amino acid accumulation, exchange and efflux (Cleal and Lewis, 2008; Lager and Powell, 2012), as well as those carrier proteins implicated in ion transportation (Gallou-Kabani et al., 2010; Gabory et al., 2012). Thus, akin to glucose, the capacity of the placenta to transport amino acids is sensitive to changes in maternal nutrient and oxygen availability during pregnancy.

Although less studied compared to amino acid and glucose, the capacity of the placenta for fatty acid uptake and transfer to the fetus is also modified by the maternal environment. In mice and rats fed high-fat diets during pregnancy, the abundances of lipoprotein lipase, fatty acid binding protein, very low-density lipoprotein receptor and/or fatty acid transporter protein are increased in the placenta near term, depending on the content of fat in the diet and whether simple sugars were additionally consumed in excess (Rebholz et al., 2011; Qiao et al., 2012, 2015; Mazzucco et al., 2013; Sferruzzi-Perri et al., 2013; Reynolds et al., 2015). Moreover, placental uptake and fetal accumulation of maternally supplied triglycerides is increased in mice fed high-fat diets (Rebholz et al., 2011). Combined, these studies indicate that placental fatty acid transport capacity is enhanced by excess maternal dietary fat, and is consistent with the increased accumulation of fat within the fetus (Mazzucco et al., 2013; Qiao et al., 2015). Placental expression of genes involved in materno-fetal lipid transfer is also increased in undernourished mice and may represent a compensatory attempt to maintain the fetal nutrient balance (Chen et al., 2013). However, further work is required to determine whether nutritional scarcity and reduced maternal oxygenation affects the lipid transport capabilities of the placenta. Moreover, the environmental regulation of materno-fetal nutrient transfer via the endocytosis-exocytosis pathway requires investigation. A need for this is reflected by observations indicating that substrate uptake via endocytosis is increased in trophectoderm cells and the yolk sac during early mouse development in response to maternal protein restriction (Watkins et al., 2008; Sun et al., 2014).

## Regulation of other essential placental functions by the maternal environment

The placenta secretes hormones that modulate maternal adaptations to pregnancy, with consequences for resource allocation to the fetus. As mentioned previously, many of the environmental manipulations in mice, rats and guinea pigs affect the volume fraction and/or volume of the placental endocrine region, which will have implications for absolute endocrine output into the mother (Table 1). Furthermore, the expression of individual hormones including, prolactin-related genes, pregnancy-specific glycoproteins, angiotensinogen, appetite regulating peptides and cytokines, is also altered by maternal nutrition and/or the oxygen supply (Ain et al., 2004; Ho-Chen et al., 2006; Caminos et al., 2008; Rosario et al., 2008; Schulz et al., 2012; Chen et al., 2013; Mazzucco et al., 2013; Cuffe et al., 2014b; George et al., 2014; Wang et al., 2015). In addition, the ability of the placenta to act as a barrier to circulating maternal hormones as well as xenobiotics, is affected by the environment of the mother (Table 1). For instance, dietary manipulation and inhalation hypoxia in rodents changes the placental expression of enzymes like 11β-hydroxysteroid dehydrogenases type 1 and 2 that activate and inactivate circulating glucocorticoids, respectively, with implications for fetal growth and maturation (Lesage et al., 2001; Belkacemi et al., 2011a,b; Gao et al., 2012a,b, 2013; Vaughan et al., 2012b, 2013, 2015a,b; Cuffe et al., 2014a; Bellisario et al., 2015a,b). Moreover, both a high-fat diet in mice and undernutrition in guinea pigs diminishes the abundance and/or activity of protective transporter proteins in the placenta, including p-glycoprotein and other ATP-binding cassette (ABC) drug efflux proteins which would have consequences for fetal exposure to endogenous and exogenous substances in the maternal circulation (Soo et al., 2012; Wang et al., 2015). Further work is required to elucidate the environmental regulation of alternate, essential placental functions in the context of fetal development and growth.

Thus, the maternal environment affects placental phenotype which has implications for the amount and relative proportions of specific metabolic substrates as well as growth-regulating hormones supplied to the fetus at different stages of development. Few studies have considered the implication of conceptus sex in context (Gallou-Kabani et al., 2010; Vickers et al., 2011; Gabory et al., 2012; King et al., 2013; Cuffe et al., 2014a; Reynolds et al., 2015; see Table 1). However, the interaction of conceptus sex with environmentally-induced changes in placental phenotype will be important for understanding the developmental programming of disease susceptibility beyond the womb.

## Effects of maternal environment on offspring growth

Several of the environmental challenges reported to affect placental phenotype (Table 1) have been associated with both immediate and long-term effects on offspring growth and wellbeing into adulthood (Table 2). Numerous adverse prenatal environments alter postnatal growth, but the specific effect is dependent on the timing and severity of the insult, as well as on the quality of the postnatal environment to which the offspring is exposed. Prenatal perturbations, such as calorie restriction (10–35%; Kind et al., 1999, 2002, 2003; Vickers et al., 2000; Riviere et al., 2005; Breton et al., 2009; Camm et al., 2011; Lukaszewski et al., 2011, 2013), low-protein diet (6%; Sathishkumar et al., 2009, 2012, 2015), iron restriction (Crowe et al., 1995; Lewis et al., 2001c, 2002) and uterine artery ligation (e.g., Wlodek et al., 2007, 2008; Siebel et al., 2008) in rats and guinea pigs reduce birth weight. Furthermore, offspring remain smaller through to adulthood, irrespective of the stage at which the manipulation occurs prenatally. Interestingly, exposure to 50% calorie restriction can have variable effects on postnatal growth, depending on whether pups are cross-fostered onto *ad libitum* fed dams after birth. Cross-fostering offspring exposed to calorie restriction prenatally results in a significant period of catch-up growth, and an increase in body weight in adulthood (Desai et al., 2008; Magee et al., 2008; Khorram et al., 2011, 2015; Fukami et al., 2012). Conversely leaving calorie-restricted offspring with their mothers during lactation does not appear to increase postnatal body weight (Leonhardt et al., 2002, 2003; Sebaai et al., 2002a,b, 2004; Vieau et al., 2007; Delahaye et al., 2008, 2010; Coupe et al., 2009; Laborie et al., 2011; Wattez et al., 2014), which may relate to impaired mammary function and compromised milk quality and quantity (Wlodek et al., 2005; O'Dowd et al., 2008). Hypoxia can reduce birth weight in rodents if exposure is during the latter third of pregnancy and is below 13% inspired oxygen, with body weight normalizing by adulthood (Walton et al., 2015). Gestational anemia induced by an iron-depleted maternal diet also reduces birth weight, with changes in postnatal growth rate depending on the length of maternal anemia prior to pregnancy (Crowe et al., 1995; Lewis et al., 2001c, 2002). Maternal high-fat diets often result in an increase in postnatal body weight at weaning and in adulthood, even when birth weight is decreased or unchanged, and may relate to whether the diet is continued during lactation (Howie et al., 2009, 2013; Liang et al., 2009b; Sloboda et al., 2009; Connor et al., 2010, 2012; Dudley et al., 2011; Smith et al., 2014; Mazzucco et al., 2016). In contrast, excess fructose may increase birth weight although postnatal growth of offspring may not be significantly altered and is normalized by adulthood (Vickers et al., 2011; Clayton et al., 2015; Lineker et al., 2015). Thus, alterations in the environment provided by the mother during pregnancy exert affects on offspring growth. Further work is required to determine the effect of calorie-dense diets and reduced maternal oxygen availability on mammary gland function in relation to offspring growth postnatally. Nonetheless, beyond gross weight, an environmental challenge to the mother in pregnancy and/or lactation effects the body composition of the offspring; that is the proportion of adipose versus lean mass and the absolute and relative weights of several individual organs (Desai et al., 2005b; Table 2). Alterations in offspring development and growth will have implications for whole body and organ function.

**Table 2:** **The effect of an environmental challenge on postnatal phenotype**.

**Manipulation**	**Species**	**Treated from**	**Birth and postnatal body weight**	**Postnatal age at study**	**Cohort examined**	**Postnatal phenotype**	**References**
**TOTAL CALORIE RESTRICTION**							
30%UN^ˆ^	Rat	D1-21	↓birth and postnatal weight Vickers et al., 2000 or catch-up growth Riviere et al., 2005	4 months	Male offspring	↑blood pressure, food intake, plasma insulin, leptin, Ang II, aldosterone and adiposity ↓relative kidney and liver weights ↓nephron number	Vickers et al., 2000; Riviere et al., 2005
			↓birth and postnatal weight^#^	4 months	Male offspring	Glucose intolerant Altered hypothalamic-regulatory system (POMC neurons sensitivity) ↑food intake, adiposity, plasma and adipose leptin, plasma corticosterone	Breton et al., 2009; Lukaszewski et al., 2011, 2013
35%UN	Rat	D15-20	↓birth weight followed by catch-up growth	4 months	Male offspring	↑plasma FFA ↓hepatic Akt-1, Akt-2 and PKCζ expression and skeletal muscle GLUT4 expression	Camm et al., 2011
50%UN	Rat	D1-D22	↓birth weight followed by catch-up growth at 3 weeks but normalized by 5 months	Birth to ~5 months	Male and female offspring	2 days: ↓plasma leptin and insulin Early onset of puberty ↓plasma progesterone levels (females only)	Sloboda et al., 2009; Smith et al., 2014
						5 months: ↑plasma leptin and insulin, plasma lipase and cholesterol (males only), adiposity, absolute and relative liver weight ↓plasma IGF-1, IGFBP3, hepatic IGFBP1 and IGFBP3 expression	
50%UN	Rat	D1-weaning	↓birth and postnatal weight at 3 weeks and 5 months	Birth to ~5 months	Male and female offspring	Early onset of puberty 5 months: ↑plasma leptin and insulin, adiposity, insulin resistant	Sloboda et al., 2009
50%UN^ˆ^	Rat	D10-21	↓birth weight followed by catch-up growth and ↑weight at 9–10 months	1 day to 10 months	Female and/or male offspring	1 day: ↓plasma glucose, insulin and triglyceride ↑lean mass ↓adipose FAS and HSL and ↑PPARγ_2_ expression ↓hepatic PPARα and PPARγ and ↑LPL, FAS and CRP expression ↓renal developmental and proliferation/apoptotic proteins (GFRAα1 and pERK1/2) Altered vascular ECM composition ↓branching angiogenesis, VEGF expression but ↑eNOS in microvessels ↓plasma leptin and E_2_, ↑LH ↑ovarian Ob-Rb, ↓ERα receptor and steroidogenic enzymes (HSD3B1, HSD3B2) ↓hypothalamic GnRH and ↑Ob-Rb and ERα receptor expression (females only)	Desai et al., 2005b, 2007a,b, 2008; Khorram et al., 2007a,b, 2010, 2015; Magee et al., 2008; Henry et al., 2010; Tafti et al., 2011; Fukami et al., 2012; Alves et al., 2015
						3 weeks: ↑adipocyte cell size and renal MEK1/2 expression ↓nephron number	
50%UN^ˆ^	Rat	D10-21	↓birth weight followed by catch-up growth and ↑weight at 9–10 months	1 day to 10 months	Female and/or male offspring	4–8 weeks: ↑food intake related to impaired leptin signaling and appetite-regulatory pathways in the hypothalamus (Ob-Rb and STAT3, assessed in males)	Desai et al., 2005b, 2007a,b, 2008; Khorram et al., 2007a,b, 2010, 2015; Magee et al., 2008; Henry et al., 2010; Tafti et al., 2011; Fukami et al., 2012; Alves et al., 2015
						2 months: ↑blood pressure, altered vascular ECM composition	
						3–4 months: ↑blood pressure and VEGF expression in vasculature Alterations in food preferences and behavioral-neurochemical responses to sweet food (↑TH content in OFC and/or NAcc)	
						9 months: glucose intolerant, ↑plasma glucose, insulin, triglyceride and CRP ↑adiposity (↑adipocyte cell size) ↓lean mass and relative heart, kidney, adrenal, liver, lung and brain weights ↑adipose SREBP1C, PPARγ_2_, FAS, HSL, LPL expression ↓hepatic PPARα and PPARγ and ↑SREBP1, LPL and FAS expression ↑adrenal leptin expression and altered steroidogenic enzyme expression (males: ↑CYP11A1, CYP11B2, HSD1, Ob-Ra/Ob-Rb and GCR, ↓CYP17A1 expression; females: ↑CYP11A1, ACTH-R and Ob-Ra, ↓GR and CYP17A1 expression)	
						10 months: ↓plasma insulin, altered appetite-regulatory pathways in the hypothalamus (females only: ↑NPY, AgRP and pAMPK/AMPK and ↓POMC) Estrous cyclicity disrupted with ↓number of corpora lutea and small follicles ↑plasma LH, FSH and T and ↓E_2_ ↑E_2_ ovarian receptor, ↓Ob-Rb, LH receptor and steroidogeneic enzymes (CYP11A1, HSD-3β1, CYP19A1) ↑hypothalamic GnRH and ↓Ob-Rb and ERα receptors (females only)	
50%UN	Rat	D10-weaning	↓birth weight followed by catch-up growth and ↑weight at 9 months	1 day, 3 weeks and 9 months	Male and female offspring	1 day: ↑plasma ghrelin and ↓plasma leptin, glucose and triglyceride	Desai et al., 2005a,b, 2007b
						3 weeks: ↓adiposity, pancreas and liver weights, plasma glucose and insulin ↑lean mass, relative lung and brain weight and plasma cholesterol	
						4–8 weeks: ↑food intake	
						9 months: Glucose intolerant, ↑plasma ghrelin, glucose, cholesterol ↓relative heart and kidney weights and plasma insulin	
50%UN	Rat	D14-weaning	↓birth weight in male offspring and ↓weight in both male and female offspring at 3 weeks	Birth to 1 month	Male and female offspring	3 weeks: Delayed puberty onset ↓plasma leptin, adiposity, testicular and ovarian weights ↓plasma FSH (males only) Altered plasma FSH (males↓; females↑) ↑UCP1 expression and altered development of gonadal WAT (favored acquisition of a brown-like phenotype, males only)	Leonhardt et al., 2003; Delahaye et al., 2010
50%UN	Rat	D14-weaning	↓postnatal weight	Birth to 8 months	Male offspring	1 day-2 weeks: ↓plasma CBG and leptin	Leonhardt et al., 2002; Lesage et al., 2002b; Sebaai et al., 2002a,b, 2004; Vieau et al., 2007; Delahaye et al., 2008; Coupe et al., 2009; Laborie et al., 2011; Wattez et al., 2014
						2 weeks-1 month: ↓hypothalamic POMC expression and delayed BDNF expression and cell proliferation in the hippocampus and hypothalamus	
						3–4 months: ↓plasma CBG, ↑plasma aldosterone and VP ↓absolute and ↑relative adrenal weight, ↓absolute kidney, thymus and liver weights Altered adrenal and kidney ANP receptor expression ↑adrenal POMC expression	
						3 weeks, 4 and 8 months: altered MR, GR expression in hippocampus, ↑plasma corticosterone	
						6 months: ↑blood pressure, plasma leptin, impaired glucose tolerance, increased food intake ↓locomotor activity	
						8 months: ↓plasma catecholamines ↑adrenal POMC, hypothalamic VP and adrenal GR and PC2 expression Alterations in adrenal medulla noradrenergic chromaffin cell aggregation and cholinergic innervation ↓expression of genes involved in cytoskeleton remodeling and vesicle trafficking in adrenal medulla	
10–30%UN	Guinea pig	-D28-birth	↓birth and postnatal weight (males only) ↔female offspring	Birth to 4 months	Male and female offspring	3–4 months: ↑plasma cholesterol and insulin, blood pressure, adiposity, adrenal weight and food intake ↓glucose tolerance and lean mass (males only)	Kind et al., 1999, 2002, 2003
**LOW-PROTEIN DIET**							
8% vs. 20% protein	Rat	D1-21	↔birth weight ↓postnatal weight at 2 months	Up to 6 months	Male offspring	↑blood pressure ↓mesenteric artery vasodilatory response	Brawley et al., 2003
8% vs. 20% protein	Rat	D1-21	↔birth weight ↓postnatal growth rate	1, 3 and/or 6 months	Male and female offspring	1 and/or 3 months (males only at 3 months): ↑blood pressure ↑hepatic PPARα, GR, Acyl-CoA expression (related to changes in DNA methylation and/or histone modifications) ↑cardiac PPARα and CPT-1 mRNA expression (related to changes in PPARα DNA methylation)	Lillycrop et al., 2005, 2007, 2008; Torrens et al., 2006; Burdge et al., 2007; Elmes et al., 2007, 2008; Rodford et al., 2008; Hoile et al., 2011; Slater-Jefferies et al., 2011
8% vs. 20% protein	Rat	D1-21	↔birth weight ↓postnatal growth rate	1, 3 and/or 6 months	Male and female offspring	Impaired mesenteric artery vasodilatory response, ↑aorta eNOS expression (males only) ↓mesenteric artery smooth muscle content ↓hepatic HO-1 expression (males only)	Lillycrop et al., 2005, 2007, 2008; Torrens et al., 2006; Burdge et al., 2007; Elmes et al., 2007, 2008; Rodford et al., 2008; Hoile et al., 2011; Slater-Jefferies et al., 2011
						6 months: Impaired left ventricular developed pressure recovery during reperfusion following myocardial ischaemia (males only)	
					Male offspring	3 months: Altered hepatic transcriptome (e.g. ion transport, developmental processes, response to oxidative species and steroid hormones) ↓hepatic GR1_10_ and PPARα DNA methylation, ↑hepatic PEPCK expression ↑blood pressure Impaired mesenteric artery vasodilatory response ↓aorta eNOS expression	
					Female offspring	2.5 months: ↑plasma glucose, altered hepatic transcriptome	
8% vs. 20% protein	Rat	D1-21	↔birth weight ↓postnatal growth rate	~1 to 6.5 months	Male and female offspring, F2 generation	↑blood pressure in male and female F2 offspring at 3.5 months Impaired mesenteric artery vasodilatory response at 3.5 and 6.5 months in male F2 offspring (female offspring not assessed)	Torrens et al., 2008; Hoile et al., 2011
					Male offspring, F2 generation	↓hepatic GR1_10_ and PPARα DNA methylation and ↑hepatic PEPCK expression	
					Female offspring, F2 generation	↑plasma glucose and altered hepatic transcriptome	
8% vs. 20% protein	Rat	D1-21	↔birth weight ↓postnatal growth rate	2.5 months	Female offspring, F3 generation	Altered hepatic transcriptome	Hoile et al., 2011
6% vs. 20% protein	Rat	D1-21	↓birth and postnatal weight	4 months	Female offspring	↑blood pressure and plasma testosterone ↓plasma oestradiol and aorta ERα expression	Sathishkumar et al., 2012
			Not reported	6 months	Female offspring	↑blood pressure, plasma testosterone and vascular Agtr1/Agtr2 ratio Enhanced mesenteric artery contractile response to Ang II	Sathishkumar et al., 2015
			↓birth and postnatal weight	12 months	Female offspring	↓estrous cyclicity ↑blood pressure Greater hypotensive and hypertensive response ↑mesenteric artery contractility and ↓vasodilatory responses ↓NO-mediated vascular responsivity	Sathishkumar et al., 2009
		D4-21	↓birth weight followed by catch-up growth	4 months	Male offspring	Glucose intolerant though ↑plasma insulin during glucose challenge ↑skeletal muscle IR and AS160 expression Defective phosphorylation of IRS-1 and GLUT4 translocation	Blesson et al., 2014
5% vs. 19% protein	Rat	D1-22	↓birth weight followed by catch-up growth	1 month	Male and female offspring	↑hepatic and muscle glycogen ↓hepatic oxygen uptake, mitochondrial swelling and lipid peroxidation ↑hepatic ADP/O ratio	Moraes et al., 2014
**HIGH-CALORIE DIETS**							
5.4x fat	Mouse	-D56-weaning	↑postnatal weight at 3 weeks	3 weeks and ~4.5 months	Male and female offspring	3 weeks: ↑plasma triglycerides (males only) ↑liver weight and lipid accumulation, ↓fatty acid oxidation genes	Mazzucco et al., 2016
						4.5 months: ↑liver FFA (females only), liver weight and lipid accumulation, ↓CPT1 (males only) and ACO expression	
12x fat	Mouse	-D28-weaning	↑postnatal weight at 6 months	6 and 12 months	Female offspring	6 months: ↑plasma glucose, ↓bone mineral density	Liang et al., 2009b
						12 months: ↑plasma insulin and glucose, blood pressure, trabecular spacing, ↓trabecular connectivity density	
4.8x fat	Rat	D1-weaning	↓birth weight followed by catch-up growth and ↑weight at 3 weeks and 5 months	Birth to ~5 months	Male and/or female offspring	2 days: ↓plasma leptin and insulin	Howie et al., 2009, 2013; Sloboda et al., 2009; Connor et al., 2010, 2012; Smith et al., 2014
						3 weeks: ↑plasma insulin Early onset of puberty	
						4–5 months: ↑plasma leptin and insulin, adiposity, bone mineral content, absolute liver weight, insulin resistant ↓plasma IGF-1, IGFBP3, corticosterone (males only) ↓hepatic IGFBP1 and IGFBP3 expression, ↑plasma lipase and cholesterol (males only) ↑pancreatic INS1, INS2, IRS1, IRS2, IL-1R1 and CD68 (males only) ↑plasma progesterone levels and prolonged and persistent oestrus ↓testes: body weight ratio and ↑adrenal ACTH-R	
4.8x fat	Rat	-D78-weaning	↓birth and postnatal weight at 3 weeks	Birth to ~5 months	Male and/or female offspring	3 weeks: ↑plasma insulin and food intake (to 2 months) Early onset of puberty ↑plasma progesterone	Howie et al., 2009, 2013; Sloboda et al., 2009; Connor et al., 2010
						5 months: ↑bone mineral content ↑pancreatic STAT3, TNF-α, CD68 and ↓PI3K expression	
2.5x fat	Rat	D1-weaning	↓birth weight followed by catch-up growth and ↑weight in adulthood	Birth to ~5 months	Male and female offspring	2 days: ↓plasma leptin and insulin Impaired hepatic proliferation (related to ↑expression and ↓DNA methylation of Cdkn1α, males only)	Dudley et al., 2011
						1 month: ↓liver and liver: brain weight ratio (males only)	
Excess of 20% fructose in drinking water	Rat	D1-P10	↑birth weight ↔postnatal weight at 10 days	Birth to 10 days	Male and female offspring	10 days: ↓absolute and relative liver and kidney weight ↑stomach content of leptin and ↓plasma insulin ↑hepatic triglycerides and SREBP1c expression	Vickers et al., 2011; Clayton et al., 2015
Excess of 20% fructose in drinking water	Rat	D1-P10	↑birth weight ↔postnatal weight at 10 days	Birth to 10 days	Male and female offspring	↓hepatic SIRT1 expression (females only) Alterations in the expression of genes regulating beta oxidation and the inflammasome, particularly in males	Vickers et al., 2011; Clayton et al., 2015
Excess of 10% fructose in drinking water	Rat	D1-D21	↔postnatal weight	3 months	Male and female offspring	↑retroperiotoneal adiponectin, FTO, MCP1 and TLR4 mRNA	Lineker et al., 2015
**HYPOXIA**							
12% hypoxia	Mouse	D14-19	↓birth weight of male and female offspring followed by catch-up growth	~2.5 months	Male and female offspring	Impaired mesenteric artery vasodilatory response and ↓elastin content in aorta ↑mesenteric artery stiffness and collagen content in aorta (males only)	Walton et al., 2015
13% hypoxia	Rat	D6-D20	↔birth weight	4 months	Male offspring	↓vasodilatory response of femoral arteries ↑myocardial contractility, LF/HF heart variability ratio, baroreflex gain and maximal and minimal heart rates	Giussani et al., 2012; Kane et al., 2013
**IRON RESTRICTION**							
Iron restriction	Rat	-D21/28-birth	↓birth and postnatal weight but greater growth rate between postnatal days 20 and 40	3 weeks and ~1.5 months	Not specified	3 weeks: ↓blood pressure, ↑relative heart and kidney weights	Crowe et al., 1995
						1.5 months: ↑blood pressure and relative heart and kidney weights	
Iron restriction	Rat	-D7-birth	↓birth and postnatal weight	3, 16, and 18 months	Male and female offspring	3 months: ↑blood pressure and serum ACE, ↓serum triglyceride	Lewis et al., 2001c, 2002
						16 months: ↑blood pressure and relative heart and kidney weights, glucose tolerance, ↓serum triglycerides	
**UTERINE SUBSTRATE SUPPLY**							
Uterine ligation^ˆ^	Rat	D18	↓birth weight followed by catch-up growth (catch-up growth females only)	Birth to 18 months	Male and female offspring	↔blood pressure or nephron number ↔vascular stiffness or reactivity (males only) ↔age of onset of puberty	Wlodek et al., 2007, 2008; Siebel et al., 2008, 2010; Wadley et al., 2008, 2013; Moritz et al., 2009; Mazzuca et al., 2010, 2012; Black et al., 2012; Lauritz et al., 2012; Tare et al., 2012; Gallo et al., 2012a,b; Master et al., 2014; Romano et al., 2014, 2015; Tran et al., 2015
Uterine ligation^†^	Rat	D18	↓birth weight and postnatal weight	Birth to 18 months	Male and female offspring	↑blood pressure and left ventricular mass, ↓nephron number ↓femur length, trabecular and cortical bone mineral contents, trabecular density and bone geometry measures Delayed onset of puberty, ↓plasma leptin levels at puberty onset and altered concentrations of sex steroids	Wlodek et al., 2007, 2008; Siebel et al., 2008, 2010; Wadley et al., 2008, 2013; Moritz et al., 2009; Mazzuca et al., 2010, 2012; Black et al., 2012; Gallo et al., 2012a,b; Lauritz et al., 2012; Tare et al., 2012; Master et al., 2014; Romano et al., 2014, 2015; Tran et al., 2015
						Female only phenotypes: Glomerular hypertrophy, ↑plasma creatine and renal TGF-β1, MMP-9 and collagen IV expression ↑HOMA and intramuscular triglycerides (6 months), ↓HOMA and improved insulin sensitivity (12 months) ↓relative islet and β-cell mass ↑plasma triglycerides, uterine artery stiffness and proportion of thick collagen fibers and ↓uterine artery relaxation ↑spatial memory Delayed developmental ↓cardiac PGC-1α and GLUT1 expression	
						Male only phenotypes: Impaired glucose tolerance and ↓insulin ecretion ↓skeletal muscle glycogen and mitochondrial regulators (PGC-1α, COX IV, mtTFA COX III) ↑mesenteric artery wall stiffness and impaired relaxation, ↓femoral artery relaxation ↑left ventricular Agtr_1*A*_ receptor and collagen 3 expression ↓cardiomyocyte number and ↑cardiac Agtr1a, Agtr1b, Blc2 and C-myc expression Altered sensorimotor gating function and enhanced motor function Exacerbated ↓cardiac PGC-1α and mtTFA expression	

*ACE, angiotensin converting enzyme; Acyl-CoA, acetyl coenzyme A; ACO, acyl-CoA oxidase; AgRP, Agouti-related peptide; Agtr, angiotensin II receptor; ACTHr, adrenocorticotropic hormone receptor; ADP/O, adenosine diphosphate/oxygen ratio; AMPK, activated protein kinase; Ang, angiotensin; Akt, protein kinase B; ANP, atrial natriuretic peptide; AS160, TBC1 domain family member 4; BDNF, brain derived neurotropic factor; Blc2, B-cell lymphoma 2; CBG, corticosterone-binding globulin; CD68, cluster of differentiation 68; Cdkn1α, cyclin-dependent kinase inhibitor 1α; COX, cytochrome oxidase; CPT-1, carnitine palmitoyltransferase I; CRP, c-reactive peptide; CYP, Cytochrome P450; ECM, extracellular matrix; eNOS, endothelial nitric oxide synthase; ERK, extracellular signal-regulated protein kinase; ERα, estrogen receptor α; FAS, fatty acid synthase; FFA, free fatty acids; FSH, follicle stimulating hormone; FTO, fat mass and obesity-associated protein; GFRA, growth factor receptor alpha; GLUT, glucose transporter; GnRH, gonadotropin-releasing hormone; GR1_10_, glucocorticoid receptor 1(10) promoter; GR, glucocorticoid receptor; HO, heme oxygenase; HOMA, homeostasis model assessment; HSD, hydroxysteroid dehyrodenase; HSL, hormone sensitive lipase; HSP, heat shock protein; IGF, insulin-like growth factor; IGFBP, insulin-like growth factor binding protein; INS, insulin; IR, insulin receptor; IRS, insulin receptor substrate; LF/HF, low/high frequency; LH, luteinizing hormone; LPL, lipoprotein lipase; MCP1, monocyte chemoattractant protein-1; MEK, mitogen-activated protein kinase; MMP, matrix metallopeptidase; MR, mineralocorticoid receptor; MtTFA, mitochondrial transcription factor A; NAcc, nucleus accumbens; NO, nitric oxide; NPY, neuropeptide Y; NT, nitro tyrosine; Ob-Ra, leptin receptor (short); Ob-Rb, leptin receptor (long); OFC, orbitofrontal cortex; P, postnatal; PC2, prohormone-convertase 2; PEPCK, phosphoenolpyruvate carboxykinase; PGC-1α, peroxisome proliferator-activated receptor gamma coactivator 1-alpha; PI3K, phosphoinositol-3 kinase; PKC, protein kinase C; POMC, proopiomelanocortin; PPAR, peroxisome proliferator-activated receptor; SIRT1, sirtuin 1; STAT3, signal transducer and activator of transcription 3; SREBP, sterol regulatory element binding protein; T, testosterone; TGF, transforming growth factor; TH, tyrosine hydroxylase; TLR, toll-like receptor; TNF, tumor-necrosis factor; UCP1, uncoupling protein 1; VEGF, vascular endothelial growth factor; VP, vasopressin; WAT, white adipose tissue*.

ˆ Cross-fostered to ad libitum control dam,

# dams allowed to eat ad libitum post-partum,

† *offspring cross-fostered to placentally-restricted dam*.

*Gestational age: mouse ~20 days, rats ~23 days, guinea pigs ~70 days*.

## Effects of maternal environment on offspring cardiovascular structure and function

Calorie restriction, low-protein diet, iron restriction and uterine artery ligation have all been shown to increase systolic blood pressure in adult offspring (Crowe et al., 1995; Vickers et al., 2000; Lewis et al., 2001c, 2002; Kind et al., 2002; Brawley et al., 2003; Riviere et al., 2005; Torrens et al., 2006, 2008; Elmes et al., 2007, 2008; Khorram et al., 2007a; Wlodek et al., 2008; Sathishkumar et al., 2009, 2012, 2015; Gallo et al., 2012b; Master et al., 2014; Wattez et al., 2014; Tran et al., 2015). The degree to which blood pressure is elevated can vary with the specific environmental exposure, and may be due, at least in part, to structural remodeling of offspring cardiac tissue (Crowe et al., 1995; Lewis et al., 2001c, 2002; Wlodek et al., 2008; Black et al., 2012) and blood vessels (Khorram et al., 2007b; Rodford et al., 2008; Giussani et al., 2012; Walton et al., 2015), changes in the expression of genes and miRNAs involved in cardiac energy metabolism (Slater-Jefferies et al., 2011), extracellular matrix remodeling (Khorram et al., 2007a, 2010; Wlodek et al., 2008), cardiac hypertrophy (Black et al., 2012), and mitochondrial biogenesis (Wadley et al., 2013). Alterations in the reactivity of resistance arteries to vasodilators or constrictors and/or myocardium contractility, may also contribute to elevated blood pressure in adult offspring (Brawley et al., 2003; Torrens et al., 2006; Sathishkumar et al., 2009, 2015; Mazzuca et al., 2010, 2012; Giussani et al., 2012, 2014; Tare et al., 2012; Kane et al., 2013; Walton et al., 2015). In relation to high-calorie diets in which the placental phenotype has been characterized, only one study has reported elevated systolic blood pressure in adult offspring (Liang et al., 2009b).

Blood pressure assessments have predominately been performed in male offspring. Whilst some studies have assessed blood pressure in female offspring (Elmes et al., 2007, 2008; Khorram et al., 2007a; Liang et al., 2009b), they often present with less severe cardiovascular dysfunction than males. Modulation of the renin-angiotensinogen system by estrogen may confer protection against the programming effects of prenatal insults on cardiac regulatory systems in female offspring (Ojeda et al., 2007, 2014). Moreover, male fetuses may be more sensitive to altered nutrient and oxygen supply due to their higher rate of intrauterine growth, compared to females (Clifton, 2010).

## Effects of maternal environment on offspring cerebral structure and function

In the hypothalamus, leptin receptor (Ob-Rb)-stimulated signal transducer and activator of transcription (STAT)-3 signaling is crucial in the control of feeding by leptin (Ghilardi et al., 1996). Calorie restriction (50%, day (D)10-21) has been shown to alter hypothalamic Ob-Rb gene expression and STAT3 protein expression, in the early postnatal period (Desai et al., 2007a). In addition, calorie restriction is associated with altered food preferences in adulthood, dopamine sensitivity and expression of appetite-stimulatory factors and hypothalamic responsiveness to alterations in energy status (altered genes include: neuropeptide Y (NPY), Agouti-related peptide (AgRP), proopiomelanocortin (POMC), and activated protein kinase (AMPK; Delahaye et al., 2008; Fukami et al., 2012; Lukaszewski et al., 2013; Alves et al., 2015). Together, these findings suggest an enhanced appetite drive which is consistent with offspring hyperphagia reported in many of these models, thus contributing to the increased risk of adult obesity in offspring (Vickers et al., 2000; Kind et al., 2003; Desai et al., 2007a; Breton et al., 2009; Delahaye et al., 2010).

Calorie restriction (50% of *ad libitum*) from day 14 through to weaning alters the structural development of the hypothalamus and hippocampus, by changing the production of brain-derived neurotrophic factor (BDNF) and cell proliferation during development (Coupe et al., 2009). These early modifications in cerebral structure may have long-lasting consequences on the regulation of neuroendocrine activity, energy metabolism and cognition. Uterine artery ligation, resulting in low-birth weight offspring, impairs sensorimotor gating, but enhances motor function and spatial memory in adult offspring (Lauritz et al., 2012). These data are inconsistent with reports in the literature which supports the *in utero* programming of motor dysfunction (Smart et al., 1973) and altered exploratory behavior (Almeida et al., 1996) in low-birth weight offspring. Thus, further studies are required to verify the motor and cognitive deficit in offspring from an altered prenatal environment, where the placental phenotype is known (for instance in response to gestational hypoxia, protein deprivation or excess maternal calories).

## Effects of maternal environment on offspring reproductive organs structure and function

Calorie restriction (50%, D10-21) increases ovarian expression of enzymes involved in androgen synthesis and plasma luteinizing hormone/follicle stimulating hormone (LH/FSH) concentrations, whilst reducing estrogen receptor (ERα) abundance and the number of corpora lutea in prepubescent and adult female offspring (Khorram et al., 2015). Calorie restriction from day 14 of pregnancy through to weaning also has long-term consequences for the size and histology of the genitals and plasma gonadotropin levels (Leonhardt et al., 2003). Furthermore, the age of onset of puberty and estrous cyclicity is altered in offspring exposed to maternal calorie restriction (50% UN; Leonhardt et al., 2003; Sloboda et al., 2009; Khorram et al., 2015), low-protein diet (Sathishkumar et al., 2009), high-fat diet (Sloboda et al., 2009; Connor et al., 2012), and uterine artery ligation (Romano et al., 2015). In part, these changes may be related to alterations in hypothalamic-gonadal communication, as hypothalamic gonadotropin-releasing hormone (GnRH) production and estrogen receptor expression are altered in offspring exposed to gestational maternal calorie restriction (Khorram et al., 2015). These studies indicate that the prenatal environment can alter reproductive maturation and function, which may be associated with altered fertility, thereby impacting the health and reproductive potential of future generations.

## Effects of maternal environment on offspring kidney structure and function

Calorie restriction (30% UN, D1-21 or 50% D10-21) results in a decreased nephron number in male offspring in the early postnatal period and in adulthood (Riviere et al., 2005; Henry et al., 2010). Uterine artery ligation also reduces nephron number in male and female offspring (Wlodek et al., 2008; Moritz et al., 2009), with female offspring developing compensatory glomerular hypertrophy and renal dysfunction later in life (Moritz et al., 2009). Dysregulated glial cell line-derived neurotrophic factor (GDNF) and mitogen-activated protein kinase-extracellular signal-regulated protein kinase (MAPK–ERK) signaling and increased apoptosis (Henry et al., 2010; Tafti et al., 2011), in conjunction with elevated vasopressin (Sebaai et al., 2002b), angiotensin II and aldosterone levels (Ang II; Riviere et al., 2005), may be key underlying factors in the pathogenesis of reduced offspring nephrogenesis and programmed hypertension.

## Effects of maternal environment on offspring metabolic systems

The effects of an adverse prenatal environment on metabolic systems have been extensively studied in a variety of animal models. Many of the prenatal insults report impaired glucose tolerance and/or insulin sensitivity which relate to defects in pancreatic formation and insulin production as well as changes in the expression of proteins in insulin-responsive pathways in key metabolic tissues like the liver, skeletal muscle and adipose tissue (Table 2; Siebel et al., 2010; Camm et al., 2011; Gallo et al., 2012a; Tran et al., 2012; Howie et al., 2013; Blesson et al., 2014). Moreover, calorie restriction (30% D1-12 or 50% D1-21), low-protein (16% D1-21) and high-calorie diets (2.5-times fat D1-weaning) result in hyperinsulinaemia, hyperglycaemia and/or hyperleptinaemia and increased adiposity in adult offspring (Vickers et al., 2000; Kind et al., 2003; Desai et al., 2005a, 2008; Magee et al., 2008; Breton et al., 2009; Howie et al., 2009, 2013; Lukaszewski et al., 2011; Fukami et al., 2012; Blesson et al., 2014). Furthermore, studies in these models have shown increases in hepatic triglycerides (Clayton et al., 2015; Mazzucco et al., 2016) and plasma cholesterol and triglycerides (Desai et al., 2007b, 2008; Magee et al., 2008; Smith et al., 2014). Maternal low-protein diets and uterine artery ligation also affect nutrient storage and metabolism in offspring tissues, including the liver and skeletal muscle (Wadley et al., 2008; Moraes et al., 2014). Persistent changes in hepatic (Magee et al., 2008; Clayton et al., 2015), adrenal (Khorram et al., 2011), and adipogenic (Desai et al., 2008) gene expression and DNA methylation (Lillycrop et al., 2005, 2007, 2008; Burdge et al., 2007; Dudley et al., 2011) have also been reported in these animal models, suggesting that epigenetic processes may be central to the mechanism by which the early environment can increase susceptibility to metabolic disease in later life (Lillycrop and Burdge, 2011). Increased adiposity of the fetus has been reported in guinea pig offspring following calorie restriction, which may contribute to the metabolic and cardiovascular dysfunction that these offspring develop as adults (Kind et al., 2005).

## Effects of maternal environment on other physiological systems

The prenatal environment may also alter other physiological systems in the fetus, having long-term effects on the offspring (e.g., bone development; Romano et al., 2014; Anevska et al., 2015) and the hypothalamic-pituitary axis (Lesage et al., 2002b). However, the sensitivity of individual organs and tissues to a prenatal challenge will likely reflect the specific substrate demands of that organ and its tempo of development. Several experimental studies report that hypoxia during pregnancy decreases maternal food intake (Camm et al., 2010; Higgins et al., 2015), and likewise undernutrition in pregnancy has been reported to cause reductions in uterine blood flow which would also deprive the conceptus of oxygen (Ahokas et al., 1983). Thus, the impact of prenatal nutrition and oxygen availability are inter-related and will together determine the phenotype of the offspring. Furthermore, the interaction of the pre-and postnatal environments is also important in determining the specific postnatal profile of the offspring. Cross-fostering pups after birth, thereby altering the lactational environment (Wlodek et al., 2005; O'Dowd et al., 2008), or varying the diet after weaning (e.g., Howie et al., 2009; Connor et al., 2010, 2012), exposes the offspring to a “second hit” and may exacerbate the effects of an adverse prenatal challenge on the phenotype of the offspring. Poor maternal care and bonding may also impact the early post-weaning phenotype of the offspring, contributing to later life physiology and disease risk (Connor et al., 2012). The postnatal environment therefore needs to be considered when reviewing the phenotypic changes observed in adult offspring. Clearly the maternal environment can impact offspring metabolism and health, though emerging evidence suggests that offspring may also prematurely age (with altered expression of senescence markers; Tarry-Adkins et al., 2009) and programming effects may extend to subsequent generations (e.g., Burdge et al., 2007; Hoile et al., 2011).

## Summary

**Figure 1:**
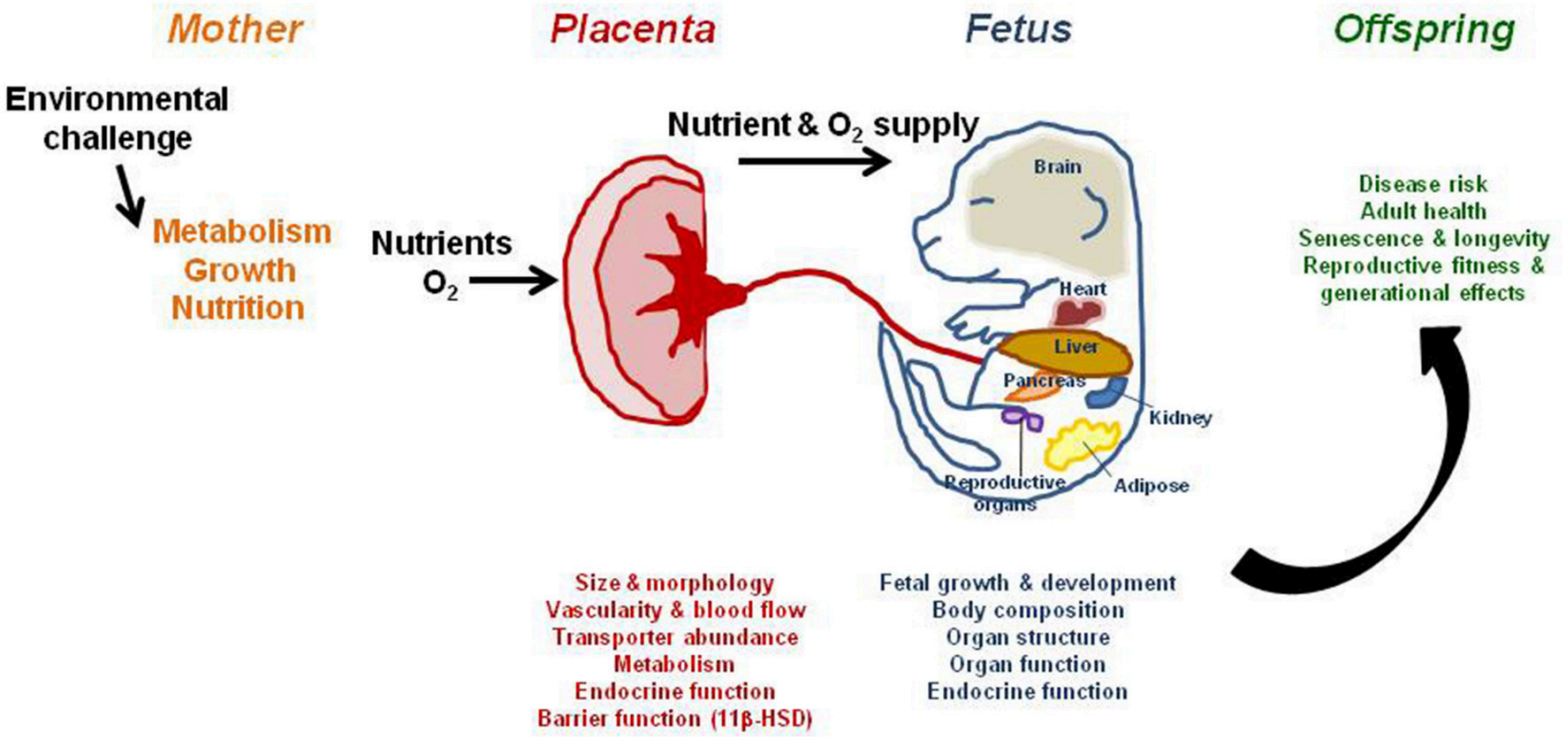
**The effect of an environmental challenge, such as maternal nutrient manipulation or oxygen scarcity, on placental phenotype, fetal growth, and offspring outcome**. The placenta can adapt morphologically and functionally to optimize substrate supply and fetal growth in the face of an environmental challenge. While these adaptations may meet the fetal drive for growth, they alter the amount and relative proportions of specific metabolic substrates supplied to the fetus during development. This will ultimately program physiological systems at the gene, cell, tissue, organ, and system levels and cause permanent structural and functional changes, leading to overt disease in adulthood, particularly with increasing age.

The maternal environment clearly affects placental and fetal growth and the postnatal phenotype of offspring (Figure 1). Identifying the postnatal functional consequences arising from an adverse prenatal environment with a known placental phenotype could aid in the development of placental biomarkers for early diagnosis, assist in identifying susceptible individuals at risk for adult disease, and contribute to the discovery of novel therapeutic strategies to prevent or ameliorate programmed effects. Indeed, placental phenotypic traits have been associated with various diseases in humans postnatally, including insulin resistance, hypertension, heart disease, asthma, cancers, as well as premature death (Barker et al., 1993, 2010a,b,c, 2011, 2012, 2013a,b,c; van Abeelen et al., 2011).

## Author contributions

ANS and EJC contributed equally to reviewing the literature and writing and editing the manuscript.

## Funding

ANS is supported by a Royal Society Dorothy Hodgkin Fellowship.

### Conflict of interest statement

The authors declare that the research was conducted in the absence of any commercial or financial relationships that could be construed as a potential conflict of interest.
